# Error in Drug Name

**DOI:** 10.1001/jamanetworkopen.2024.56401

**Published:** 2024-12-20

In the Invited Commentary titled “Respiratory Syncytial Virus and United States Pediatric Intensive Care Utilization—A New Era?,”^1^ published October 25, 2024, Pfizer’s bivalent RSV maternal vaccine was referred to by the wrong brand name; it should have been “Abrysvo.” This article has been corrected.^1^
